# CRISPR-mediated genotypic and phenotypic correction of a chronic granulomatous disease mutation in human iPS cells

**DOI:** 10.1016/j.exphem.2015.06.002

**Published:** 2015-10

**Authors:** Rowan Flynn, Alexander Grundmann, Peter Renz, Walther Hänseler, William S. James, Sally A. Cowley, Michael D. Moore

**Affiliations:** a James Martin Stem Cell Facility, Sir William Dunn School of Pathology, University of Oxford, Oxford, United Kingdom; b Sir William Dunn School of Pathology, University of Oxford, Oxford, United Kingdom

## Abstract

Chronic granulomatous disease (CGD) is a rare genetic disease characterized by severe and persistent childhood infections. It is caused by the lack of an antipathogen oxidative burst, normally performed by phagocytic cells to contain and clear bacterial and fungal growth. Restoration of immune function can be achieved with heterologous bone marrow transplantation; however, autologous bone marrow transplantation would be a preferable option. Thus, a method is required to recapitulate the function of the diseased gene within the patient's own cells. Gene therapy approaches for CGD have employed randomly integrating viruses with concomitant issues of insertional mutagenesis, inaccurate gene dosage, and gene silencing. Here, we explore the potential of the recently described clustered regularly interspaced short palindromic repeat (CRISPR)-Cas9 site-specific nuclease system to encourage repair of the endogenous gene by enhancing the levels of homologous recombination. Using induced pluripotent stem cells derived from a CGD patient containing a single intronic mutation in the *CYBB* gene, we show that footprintless gene editing is a viable option to correct disease mutations. Gene correction results in restoration of oxidative burst function in iPS-derived phagocytes by reintroduction of a previously skipped exon in the cytochrome b-245 heavy chain (CYBB) protein. This study provides proof-of-principle for a gene therapy approach to CGD treatment using CRISPR-Cas9.

## Highlights

• Chronic granulomatous disease–causing mutation was corrected in patient-derived iPS cells using CRISPR-Cas9
• A key to efficiency is prevention of CRISPR activity on corrected gene
• Potentially clinically relevant efficiencies are attainable with CRISPR-Cas9
• The defect in ROS production from macrophages was overcome in patient-derived cells

The advent of site-specific nucleases has stimulated much excitement for their potential to spawn a new era of in vitro experimental human genetics, in a similar vein to the impact of transgenic mice in the 1980s. Site-specific nucleases also have great potential as therapeutic tools, in theory capable of elevating homologous recombination in human cells to a level that could truly provide a personalized curative gene therapy option for genetic diseases [1,2]. Here, we investigate the site-specific clustered regularly interspaced short palindromic repeat (CRISPR)-Cas9 system for correction of a point mutation in the *CYBB* gene that results in chronic granulomatous disease (CGD).

CGD, a disease characterized by recurrent, severe bacterial and fungal infections, results from an inability of phagocytic cells, particularly the innate immune sentinels macrophages and neutrophils, to generate an oxidative burst upon recognition of an invading pathogen [3]. This oxidative burst generates various reactive oxygen species (ROS), such as hydrogen peroxide, that are able to neutralize the pathogen, thereby aiding in clearance and preventing its continued spread. Although antibiotic treatment options exist for CGD, they are not optimal, since there is a lifelong dependency, and the only curative therapy involves heterologous bone marrow transplantation, which has its own inherent risks. Human leukocyte antigen (HLA)-identical donors outside siblings are also extremely rare. An alternative treatment option, gene therapy using autologous bone marrow transplantation of hematopoietic stem cells modified with retroviral vectors to express a wild-type (WT) copy of the mutated gene, has been attempted in clinical trials, with initial curative success [4]. However, the expression of the transgene waned with time, and complications arose due to insertional mutagenesis resulting in myelodysplasia [5]. This demonstrates the potential for success but also the need for a cleaner system to perfectly genetically correct the diseased genome.

Homologous recombination as an experimental tool has historically been an inefficient process, the use of which has been constrained to a limited range of model organisms (notably bacteria, yeast, trypanosomes, and transgenic mice [6–8]). The development of site-specific nucleases, such as that based on the bacterial adaptive antiviral immune system, CRISPR-Cas9 [9], have been key in expanding the use of homologous recombination in human cells. Creation of double-strand breaks (DSBs) at the precise location desired for genetic modification can enhance the efficiency of homologous recombination to levels that allow both easy isolation of modified cells and, depending on requirement, the use of the cells as a mixed population of modified and unmodified cells [10].

CGD is a monogenic disease and is a prime candidate for gene therapy, particularly since bone marrow transplantation is already a treatment option. Although there are a number of genes involved in the ROS-producing nicotinamide adenine dinucleotide phosphate (NADPH) oxidase complex, the mutation of any of which can result in CGD, the majority of cases (>60%) are due to loss of function of the cytochrome b-245 heavy chain (CYBB) protein (or GP91^PHOX^) [11]. The gene encoding CYBB is located on the X chromosome and, therefore, is only present as a single copy in male sufferers. We [12] and others [13] have previously generated induced pluripotent stem cells from CGD suffers, the differentiated myeloid descendants of which recapitulate the ROS defect characteristic of the disease. Using one of these patient-derived iPS cell lines (CGD2) with a single point mutation (T > G) at the end of intron 1 of *CYBB* [12], we report high levels of gene correction using CRISPR-Cas9, show recovery of gene function in differentiated phagocytic progeny cells, and demonstrate that the T > G mutation results in exon skipping within the mRNA of CYBB.

## Materials and methods

### Cell culture

Cell culture reagents were sourced from Invitrogen unless otherwise stated. Wild-type human iPS cell lines NHDF1 [14] and OX1-19 [15], as well as CGD-patient-derived iPS cells CGD1 (iPSC-CGD1.1 containing a frameshift mutation in exon 2 of the *P47*^*PHOX*^ gene) [12] and CGD2 (iPSC-CGD2 containing point mutation in intron 1 of the *CYBB* gene) [12], have been characterized previously and were collected with informed consent and ethical approval (REC 10/H0505/71 and Zurich 2010-0077/2, respectively). IPS cell lines were grown in mTeSR1 on Matrigel (Corning)-coated tissue culture dishes, passaged using TrypLE, and plated with the Rho-kinase inhibitor Y-27632 (10 μmol/L; Abcam). 293 and 293T cells were grown in Dulbecco's modiﬁed Eagle's medium (DMEM) containing 10% fetal calf serum (FCS), 100 U/mL penicillin, and 100 μg/mL streptomycin (D10).

### Vector construction

The CRISPR-Cas9 vectors used in this study were based on the dual Cas9-and guide RNA (gRNA)-expressing, pX330 plasmid, the Cas9^D10A^-expressing-derivative, pX335, and its puromycin-resistance gene-expressing derivative, pX462 [16] (gifts from Feng Zhang; Addgene plasmids #42230, #42335, and #48141). Cloning was performed as previously described [16] using oligonucleotides Crisprgp912f (CACCGAAACTTCATGCAATTATTT) and Crisprgp912r (AAACAAATAATTGCATGAAGTTTC) with pX335 to create pX335-gp912; oligonucleotides Crisprgp913f (CACCGCAGTAGATTCCACACAAGAC) and Crisprgp913r (AAACGTCTTGTGTGGAATCTACTGC) with pX462 to create pX462-gp913; and oligonucleotides RF178 (CACCGTCGTGCTGCTTCATGTGGT) and RF179 (AAACACCACATGAAGCAGCACGAC) with pX462 to create pX462-g27. The blue fluorescent protein (BFP)-expressing vector (pHR'SIN-cPPT-EF1-BIP) was created by site-directed mutagenesis of the *eGFP* gene present in the original pHR'SIN-cPPT-EF1-GIP (a derivative of pHR'SIN-cPPT-SE [17], but containing enhanced green fluorescent protein [eGFP] and puromycin resistance gene expressed from an internal EF1α promoter), using primers pGChismut5 (CACCTGAGCCACGGCGTGCAGTGCTT) and pGChismut6 (GCACGCCGTGGCTCAAGGTGGTCACGAG). The gene-editing donor vectors were constructed using standard TOPO cloning (Invitrogen) of polymerase chain reaction (PCR)-amplified product of genomic DNA from OX1-19 cells using CYBB-specific primers (forward GAATGGAATATGAATGGAGCTTTTG, reverse CCTGTATCCATCCATCAACTCATCT) to create pTOPO-gp91, as well as pHR'SIN-cPPT-EF1-GIP using specific primers (forward GGGGAGAACCGTATATAAGTGCAG, reverse GCCTAGACGTTTTTTAACCTCGACT) to create pTOPO-GFP (WT Plasmid). Site-directed mutagenesis using primers RF180 (CAGCCGCTACCCGGACCACATGAAG) and RF181 (CTTCATGTGGTCCGGGTAGCGGCTG) was performed on pTOPO-GFP to remove the protospacer-adjacent motif (PAM) of gRNA-27 and create Mut plasmid. All vectors were sequenced to ensure sequence integrity.

### Lentiviral transduction and transfection

Vesicular stomatitis virus G protein (VSV-g) pseudotyped lentiviral vectors, generated in 293T cells using pHR’SIN-cPPT-EF1-BIP, pCMV-deltaR8.2, and pMD2.G (gifts from Didier Trono; Addgene plasmids #12263 and #12259), were used to transduce OX1-19 and 293 cells at low multiplicity of infection (MOI; 0.15), to ensure only a single integrant per cell. Following puromycin selection, the resulting OX1-19.BIP and 293.BIP cell lines were used to quantify gene-editing frequencies by transfection. DNA transfection of 293.BIP cells was performed using TurboFect (ThermoScientific) according to the manufacturer's protocol. Briefly, 4 × 10^5^ cells plated the day before transfection were transfected with 1 μg total DNA (0.35 μg pX462-g27, 0.35 μg WT or Mut plasmid, and 0.4 μg pCAG-dsRED [a gift from Connie Cepko; Addgene plasmid # 11151] [18]). Three days posttransfection, the cells were harvested for quantification of transfection efficiency by flow cytometry for dsRED expression and replated for another 2 days; then eGFP expression was quantified by flow cytometry. The OX1-19.BIP cells were transfected in a single-cell suspension by electroporation (neon transfection platform, Invitrogen), using 2 × 10^5^ cells in a 10 μL tip with 1 μg total DNA (0.5 μg pX462-g27 plasmid and 0.5 μg WT or Mut plasmid). For ssODN transfections, 1 μg total DNA was transfected (0.5 μg pX462-g27 plasmid and 0.5 μg ssODN) using WT ssODN (GTCGTCCTTGAAGAAGATGGTGCGCTCCTGGACGTAGCCTTCGGGCATGGCGGACTTGAAGAAGTCGTGCTGCTTCATGTGGTCGGGGTAGCGGCTGAAGCACTGCACGCCGTAGGTCAGGGTGGTCACGAGGGTGGGCCAGGGCACGGGCAGCTTGCCG) and Mut ssODN (GTCGTCCTTGAAGAAGATGGTGCGCTCCTGGACGTAGCCTTCGGGCATGGCGGACTTGAAGAAGTCGTGCTGCTTCATGTGGTCCGGGTAGCGGCTGAAGCACTGCACGCCGTAGGTCAGGGTGGTCACGAGGGTGGGCCAGGGCACGGGCAGCTTGCCG). After one pulse of electroporation at 1400 volts, 20 msec pulse width, the cells were plated onto Matrigel in mTeSR1 containing 10 μmol/L Y-27632 without penicillin/streptomycin. Five days posttransfection, the cells were assayed for eGFP expression by flow cytometry.

### Gene editing and single-cell cloning

For gene editing at the *CYBB* locus, 1 × 10^6^ CGD2 iPS cells were transfected by electroporation (1000 volts, 40 msec pulse width, one pulse) in a 100 μL tip with 10 μg total DNA (2.5 μg pTOPO-gp91, 3.75 μg pX335-gp91.2, 3.75 μg pX462-gp91.3). After electroporation, the cells were plated at high density (5 × 10^5^ cells/cm^2^) for 24 hours before passaging into lower density with the addition of 1 μg/mL puromycin. Twenty-four hours later, the selection was removed, and surviving cells (GC16A) were assayed for gene-editing frequency by sequencing the *CYBB* locus using PCR primers gp91forward1 (GGTATACTGGCCAAATCATA) and gp91reverse4 (AATTGTTGGAGTGAGAGTCAA). The GC16 cell line was plated at low density onto mitotically-inactivated mouse embryonic feeder (MEF; outbred Swiss mice established and maintained at the Department of Pathology, Oxford [19,20]) cells on gelatin-coated tissue culture plates in hESC medium (KO-DMEM, 2 mmol/L L-Glutamine, 100 mmol/L nonessential amino acids, 20% serum replacement, and 8 ng/mL basic fibroblastic growth factor (FGF2)). Colonies were manually selected, sequenced using *CYBB*-specific PCR primers gp91forward1 and gp91reverse2 (CAGGAAGTTGCAATGGAGGGA), and converted to growth on Matrigel in mTeSR1.

### Monocyte/macrophage differentiation and ROS activity

Production of monocytes and macrophages from iPS cells has been described previously [15]. Monocytes released from the factories were either used directly, adhered to tissue-culture plates for 24 hours, or differentiated into macrophages on tissue culture plates in XVIVO 15 supplemented with GlutaMAX and 100 ng/mL macrophage-colony stimulating factor (M-CSF), before analysis for ROS production. Nitroblue tetrazolium (NBT), dihydrorhodamine (DHR), and luminol assays were used to identify ROS activity from monocytes and macrophages, as previously described [12], after stimulation with 500 ng/mL phorbol 12-myristate 13-acetate (PMA; 200 ng/mL for the luminol assay) or 500 ng/mL PMA and 0.5 μmol/L formyl-methionyl-leucyl-phenylalanine (fMLP), respectively.

### Quantitative real-time PCR (qRT-PCR)

The level of CYBB mRNA was quantified using two different primer pairs, compared with the level of the endogenous control, β-Actin (Eurogentec), and expressed as relative quantities compared with the WT cell line using the ΔΔCT method [21]. Primers used included gp91 exon 1 forward (CAACACATTCAACCTCTGCC), gp91 exon 3 reverse (GGACAGCAGATTTCGACAG), gp91 exon1-2 boundary forward (TTTTGTCATTCTGGTTTGGCTG), and gp91 exon 2-3 boundary reverse (CCAGTGCTGACCCAAGAAGT). Reactions were carried out in triplicate on an ABI StepOne Plus qPCR machine using SensiMix SYBR reagent (Bioline).

## Results

### CRISPR-Cas9 system has the potential to perform gene editing at clinically relevant levels for CGD

**Figure 1:**
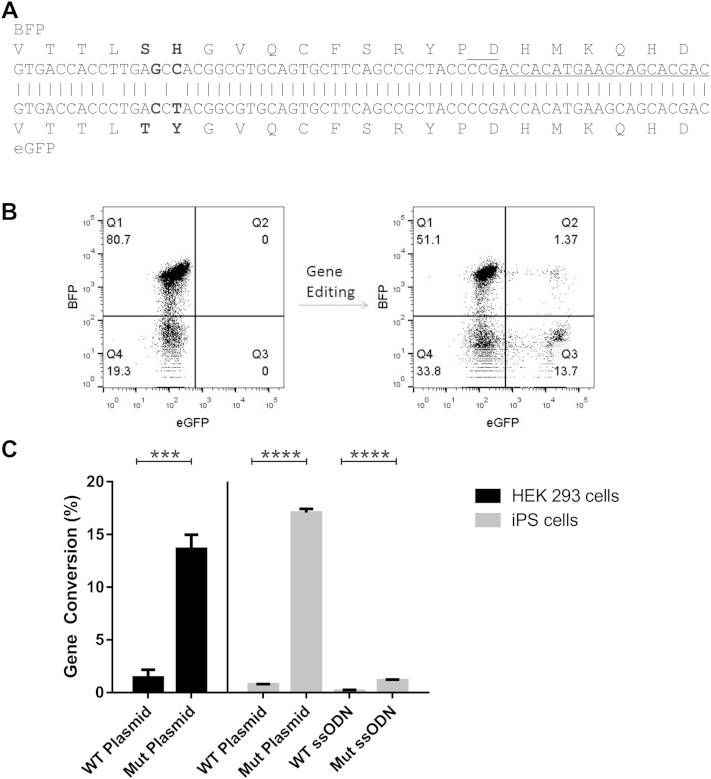
Fluorescent reporter system for CRISPR-Cas9 activity. (**A**) Alignment of *BFP* and *eGFP* showing the mutations that are required for gene editing to convert *BFP* into *eGFP* (bold), the location of gRNA-27 target site (underlined), and its PAM (overlined). (**B**) Representative dot plot showing the change in fluorescence from BFP-expressing cells to eGFP after CRISPR-Cas9 mediated gene editing in iPS cells. (**C**) Gene-editing efficiency of *BFP* into *eGFP* in HEK 293 cells (black) and OX1-19 iPS cells (grey bars) using gRNA-27 and donor templates, as indicated. Templates included plasmids containing the WT *eGFP* sequence (WT plasmid) or a mutated *eGFP* sequence with the PAM altered to prevent cleavage without altering the amino acid sequence (Mut plasmid), as well as single-stranded oligonucleotides with the WT (WT ssODN) or PAM mutant (Mut ssODN) sequences. Experiments are presented as mean ± standard deviation of three independent experiments normalized to transfection efficiency measured by DsRed cotransfected plasmid for 293 cells, and ±standard deviation of an experiment performed in triplicate for the iPS cells. Statistical significance was calculated by Student's *t* test. ***p < 0.001; ****p < 0.0001.

The frequency of cells with a functional NADPH oxidase complex required to relieve CGD sufferers' symptoms is estimated to be only 10% of circulating monocytes and neutrophils [22–24]. Thus, we set out to establish if such levels of gene editing were possible using a model system for accurate quantification of homologous recombination rates. The model consisted of cell lines (either HEK293 or OX1-19 iPS cells) transduced by a lentiviral vector expressing a BFP that differs from eGFP by two neighboring amino acid substitutions within the chromophore (S65T and H66Y; Fig. 1A). After transfection of these cells with a plasmid expressing Cas9 and a guide RNA (gRNA-27) targeting a site nearby the BFP/eGFP mutations, along with a repair template containing a partial WT eGFP sequence, the fluorescent marker switch from BFP to eGFP can be easily detected and the gene editing frequency quantified by flow cytometry (Fig. 1B). Using this model, we were able to show that the CRISPR-Cas9 system is able to achieve rates of homologous recombination above 10% in both HEK293 cells and iPS cells (Fig. 1C). This rate of gene editing (17.0% ± 0.35 for iPS cell and 13.6% ± 1.4 for HEK293 cells) was only observed when using a plasmid donor template (Mut plasmid) containing a mutated PAM, a sequence essential for cleavage by Cas9. In contrast, without a mutated PAM (WT plasmid) tenfold lower levels of gene editing were detected (0.77% ± 0.05 for iPS cells and 1.4% ± 0.75 for HEK293 cells). A similar phenomenon was observed when using single-stranded oligonucleotides (ssODNs) as donor templates; an ssODN with a mutated PAM (Mut ssODN) was more efficient than an ssODN with the WT sequence (WT ssODN; 1.16% ± 0.09 versus 0.16% ± 0.1; *p* < 0.001; Fig. 1B). Since the ssODN was noncomplementary to the gRNA-27 and, therefore, not a target for cleavage by Cas9, the reduction in gene editing observed with a WT PAM was likely due to Cas9-directed cleavage and mutation of the gene after successful homologous recombination, resulting in loss of the eGFP signal. Moreover, in our hands the level of gene editing using plasmid donors far exceeded that of single-stranded oligonucleotides, and, therefore, a plasmid donor was chosen for correction of the CGD-causing mutation.

### Gene editing of the endogenous *CYBB* gene locus using the CRISPR-Cas9 system can reach clinically relevant levels

**Supplementary Figure E1:**
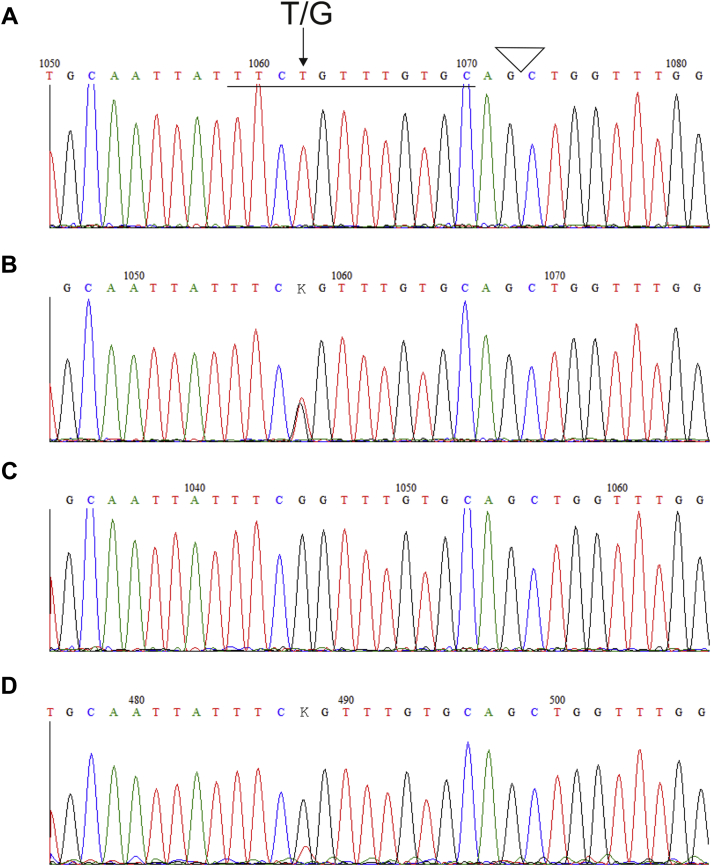
The heterogeneous pool of CRISPR-gRNA–transfected cells shows high levels of gene editing at the *CYBB* locus. Different ratios of WT and mutant plasmids were sequenced: (**A**) 100% WT, (**B**) 40:60 WT/mutant, and (**C**) 100% mutant. (**D**) This provided a standard curve of signal intensities at the T/G mutation site and allowed an estimate of the efficiency of gene conversion within a heterogeneous pool of gene-converted cells. The location of the G > T mutation is highlighted in (**A**), along with the polypyrimidine tract (underlined) and the splice site (inverted triangle). WT = wild type.

**Figure 2:**
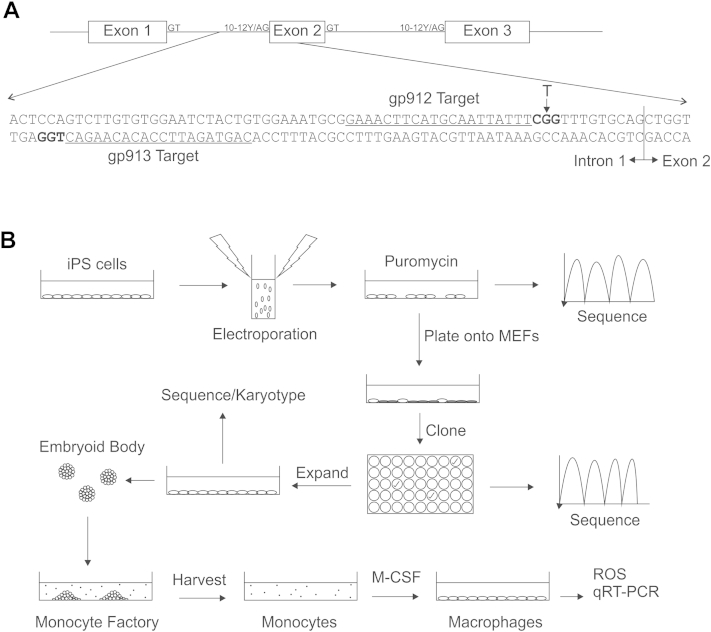
Schematic representation of the *CYBB* gene correction protocol. (**A**) Genomic organization of the first three exons of *CYBB* with consensus splice donor (GT), splice acceptor (AG) and polypyrimidine tract (10-12Y) shown for each intron. An enhanced view showing the relevant segment of the intron 1/exon 2 boundary is also shown to highlight the location of the CGD2 patient's T > G mutation within the polypyrimidine tract and the location of the two CRISPR-gRNA target sites (gp912 and gp913, underlined), along with their respective PAMs (bold). (**B**) Induced pluripotent stem cells generated from patient CGD2 were grown under feeder free conditions, electroporated with CRISPR-gRNA constructs and repair template, and selected for transfected cells using puromycin. The residual heterogeneous population of cells was assayed for gene-editing frequency by sequencing and was subsequently passaged onto MEF feeder cells. Single-cell clones were picked, grown on MEFs, and assayed individually for their sequence at the *CYBB* gene. Successfully modified clones were expanded, converted to feeder-free culture, assayed for pluripotency and intact karyotype, and differentiated into monocytes/macrophages using an embryoid-body-based protocol [15]. Monocytes harvested from the factory supernatant, and subsequently M-CSF-differentiated macrophages, were then assayed for phenotypic correction of the CGD mutation.

With the aim being establishment of protocols for gene therapy of CGD using the CRISPR-Cas9 system, we chose to make use of the D10A-mutated version of Cas9, which is only capable of cleaving a single strand of DNA [25]. By providing two neighboring gRNAs, the Cas9^D10A^ protein can create a DSB, resulting in similar levels of homologous recombination to the WT Cas9, but with the added advantage of increasing the specificity of DSB formation and, therefore, reducing off-target mutagenic events [25]. The location of the gRNAs selected to target the *CYBB* gene of iPS cells from patient CGD2 are shown in Figure 2A. Note that one of the gRNA target sites contains the disease-causing mutation within its PAM, which is not present within the WT *CYBB* sequence; our intention was to improve the efficiency of gene editing as seen in the fluorescent reporter model. Using these conditions, gene editing at the *CYBB* locus was detected at frequency of 11% (CGD2.GC16A cell pool), as measured by the relative signal intensities of the mixed chromatograms of the sequencing reaction (Supplementary Figure E1, online only, available at www.exphem.org).

**Supplementary Figure E2:**
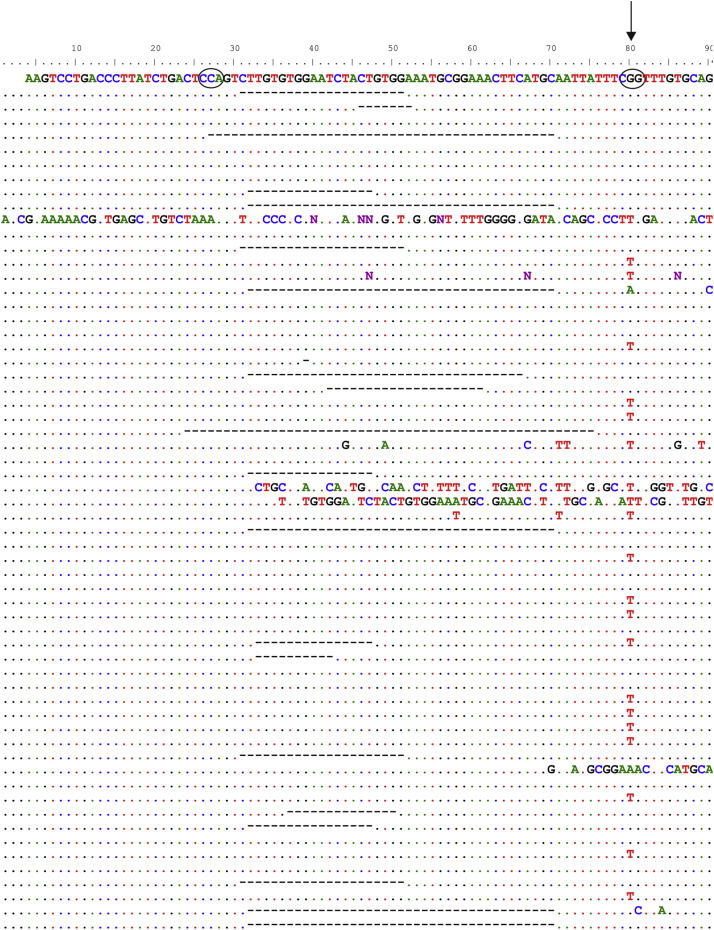
Sequence analysis of single-cell clones derived from the heterogeneous pool of gene-converted cells shows gene editing to be a frequent event. The *CYBB* loci of 60 single-cell clones were aligned to the parental cell line CGD2 (shown at the top with PAM sites circled and an arrow indicating the G > T mutation). Deletions induced by non-homologous end joining (NHEJ) following the CRISPR-gRNA–directed DSB are shown with dashes.

**Supplementary Figure E3:**
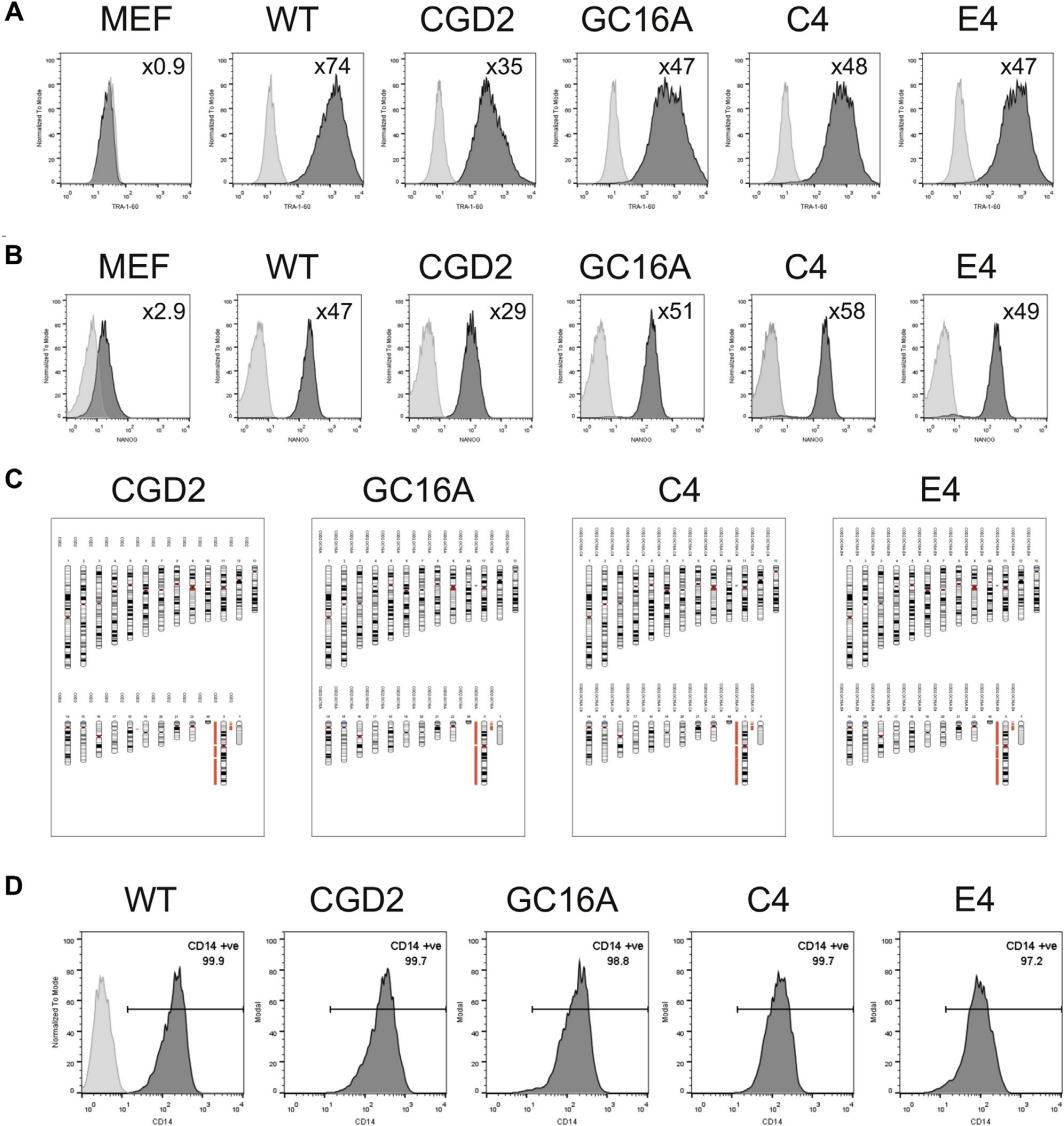
Gene-edited single-cell clones maintain pluripotent marker expression, maintain a normal karyotype, and are able to produce monocytes. iPS cells were stained for the pluripotent markers (**A**) TRA-1-60 (1.5 mg/mL; Biolegend) and (**B**) NANOG (0.3 mg/mL; Cell Signaling Technologies). Histogram plots are shown with antibody staining (dark gray) compared with their respective isotype controls (light gray); inset is the fold change in mean fluorescent intensity (MFI) of antibody to isotype. Also shown are negative control cells (MEFs). Cell labeling was quantified by flow cytometry on a BD FACSCalibur. (**C**) DNA extracted from the iPS cells was karyotyped using a SNP array (Illumina OmniExpress24 chip covering ∼700,000 markers) and analyzed using KaryoStudio (Illumina) to detect copy number variations across the genome. Red indicates a single copy of the SNPs (demonstrated by the single X copy in this male patient's DNA); gray indicates loss of heterozygosity; and green indicates duplications of a stretch of DNA (none present). (**D**) Cells harvested from the monocyte factories were stained for CD14 (Immunotools) to ensure that they were of the monocyte/macrophage lineage. Histograms from one harvest of cells are shown, indicating the percentage of live cells positive for CD14 (shown on gate) based on an unstained wild-type monocyte population (light gray).

Single cell clones, generated from CGD2.GC16A cells, were sequenced for their *CYBB* identity (Supplementary Figure E2, online only, available at www.exphem.org). Of the 60 isolated clones, 14 (23%) were correctly modified, containing the WT sequence at the intron/exon boundary, 21 (35%) contained insertion or deletion mutations at the site of CRISPR-Cas9 cleavage, and 18 (30%) were unaffected by the treatment. Thus, in agreement with our fluorescent model, levels of gene conversion that have the potential to be clinically relevant were obtained using CRISPR-Cas9 gene editing at the *CYBB* locus. Two clones (C4 and E4) were expanded and assayed for pluripotency (Supplementary Figures E3A and E3B, online only, available at www.exphem.org) and karyotypic abnormalities (Supplementary Figure E3C, online only, available at www.exphem.org) to ensure that the procedure had not adversely affected their potential to generate phagocytic cells and to serve as isogenic cell lines for the parental unmodified CGD2 cell line.

### Gene correction results in recovery of ROS activity

To demonstrate phenotypic correction at the *CYBB* locus, as well as genotypic correction, the iPS cells were differentiated into monocytes using an embryoid body-based protocol developed in our laboratory [15,26]. As was observed previously [12], NADPH-oxidase-positive and -negative lines all differentiated with similar efficiency, and monocyte factories from all cell lines produced nonadherent single cells that were over 95% CD14 positive, as determined by flow cytometry (Supplementary Figure E3D, online only, available at www.exphem.org). To act as controls for subsequent experiments, alongside the CGD2 cell lines and its derivatives, monocytes were differentiated from WT iPS cells as well as from a CGD patient (CGD1) with a mutation in the P47^Phox^ subunit of the NADPH oxidase complex, which completely abolishes ROS production [12].

**Figure 3:**
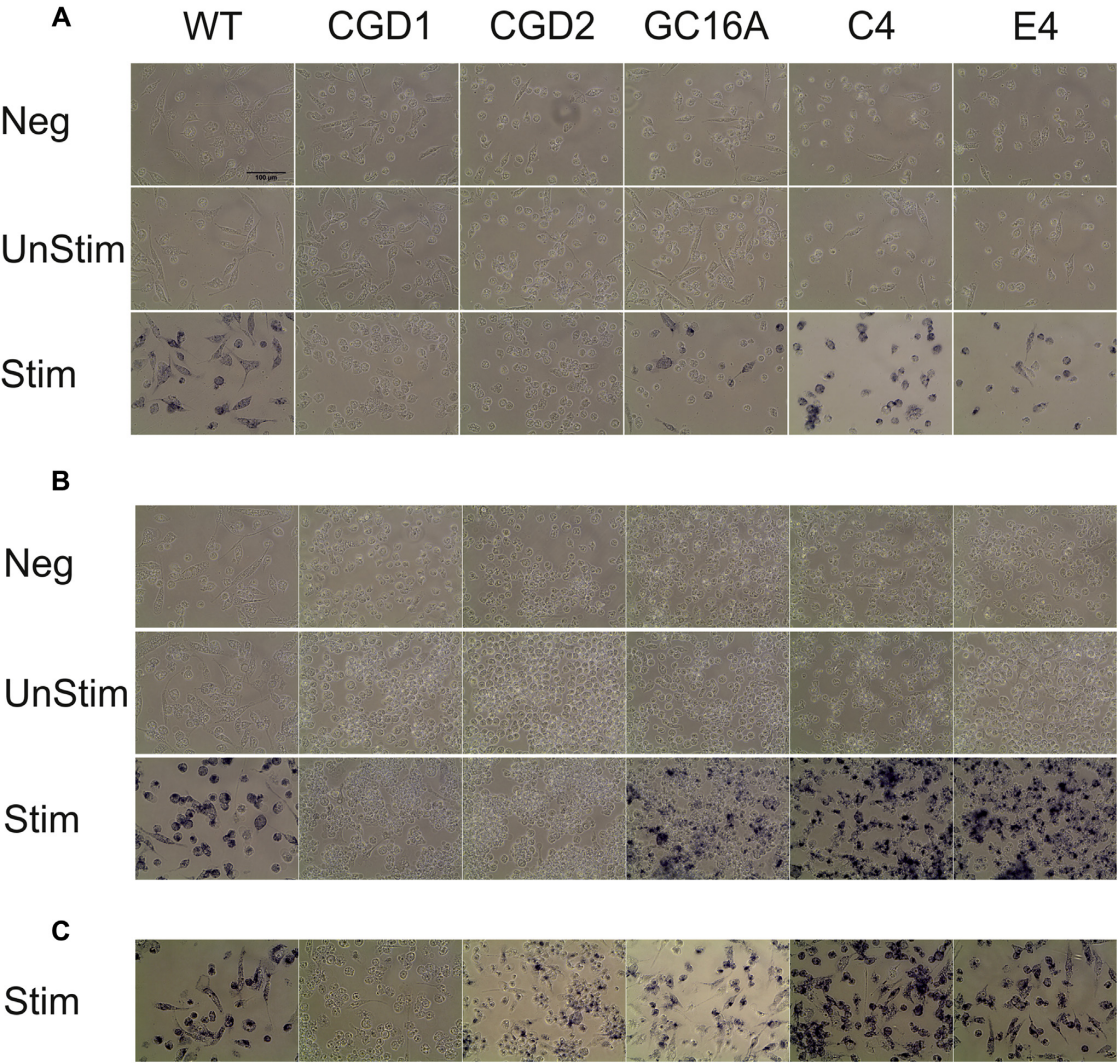
Correction of *CYBB* provides CGD2 iPS-derived myeloid cells with the ability to produce ROS. (**A**) Monocytes, (**B**) macrophages from WT NHDF1 iPS cells (WT), a *P47^Phox^* mutant iPS cell line (CGD1), a *CYBB* mutant iPS cell line (CGD2), the mixed pool CGD2.GC16A of gene-edited iPS cells (GC16A), and the two gene-edited single-cell clones from CGD2.GC16A (C4 and E4) were stimulated with PMA or with PMA and fMLP, respectively, in the presence of NBT for 1 hour. Following exposure, the cells were washed in phosphate-buffered saline and fixed. Brightfield images were taken of cells not exposed to the stimulant or the NBT (Neg), cells exposed to the NBT but without stimulation (UnStim), and cells exposed to both NBT and stimulant (Stim). Images are representative of two independent experiments. (**C**) Macrophages were incubated for an additional 1 hour with the NBT and stimulant to show the very low residual level of ROS production within the parental CGD2 cell line. Active ROS production can be seen by the precipitations of dark ROS-mediated reduced NBT. Images were taken on an EVOS inverted microscope; scale bar = 100 μm.

Three different assays for the generation of ROS activity were performed on the iPS-derived myeloid cells. Firstly, a qualitative assay for ROS, the NBT assay, was carried out on monocytes adhered to tissue culture-treated plastic for 24 hours (Fig. 3A), and also on monocytes differentiated into macrophages for 7 days in M-CSF-containing medium (Fig. 3B). The NBT assay relies on the reduction of the soluble yellow NBT substrate into a colored precipitate by the action of ROS. Wild-type cells were able to generate ROS upon stimulation, whereas the negative control line, CGD1, was not (Figs. 3A and 3B). As expected, phagocytic cells from the CGD2 cell line appeared negative for ROS by the NBT assay. The mixed population of gene-edited cells, CGD2.GC16A, showed cells staining positive for ROS, and the single-cell clones (CGD2.GC16A.C4 and CGD2.GC16A.E4) derived from CGD2.GC16A all stained positive, showing highly effective phenotypic correction of the ROS defect in cells derived from the CGD patient. As noticed previously [12], a very low level of residual NADPH oxidase activity is present in CGD2 cells, which only becomes apparent upon extended incubation of the cells with the NBT reagent for an additional hour (Fig. 3C).

**Figure 4:**
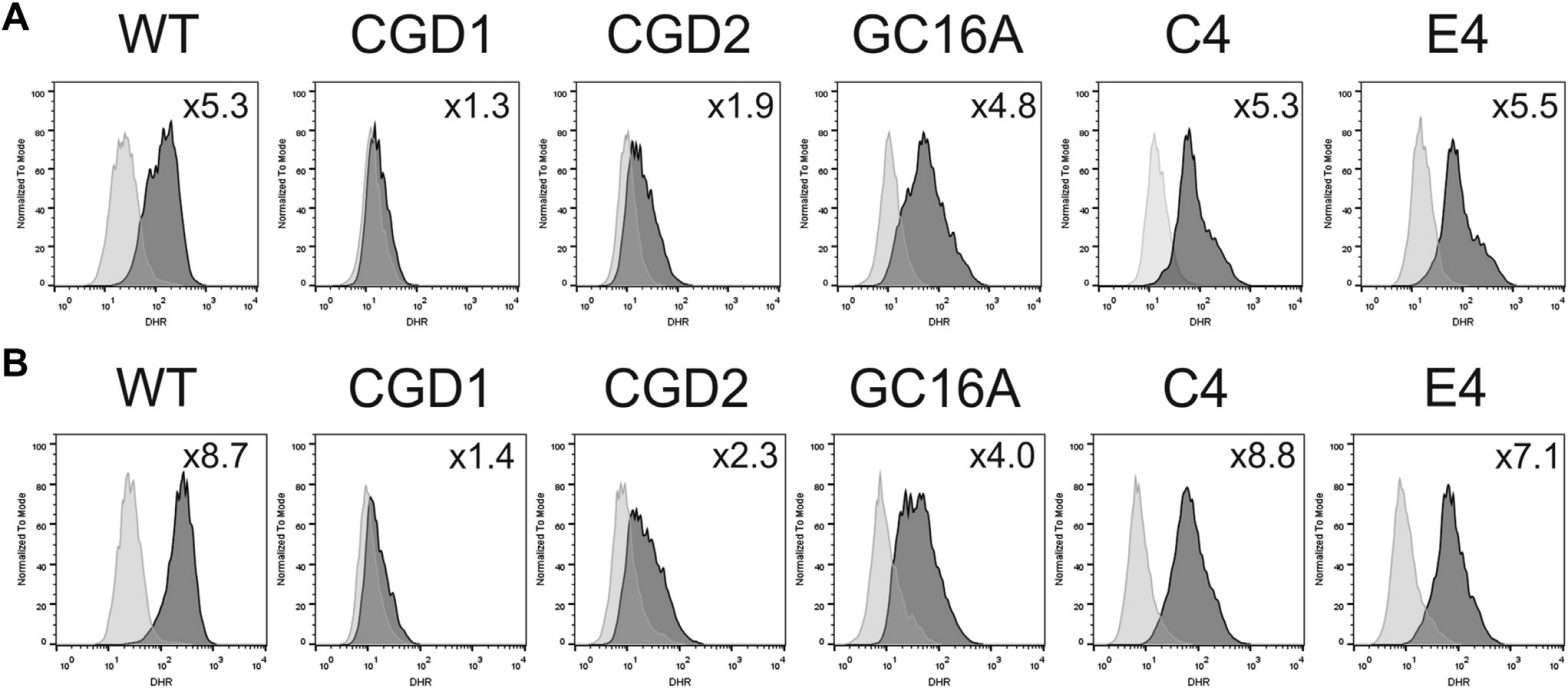
Quantification of ROS in *CYBB*-gene-corrected cell lines, as detected by DHR assay. (**A**) Monocytes and (**B**) macrophages from WT NHDF1 iPS cells (WT), a *P47^Phox^* mutant iPS cell line (CGD1), a *CYBB* mutant iPS cell line (CGD2), the mixed pool CGD2.GC16A of gene edited iPS cells (GC16A), and the two gene-edited single-cell clones from CGD2.GC16A (C4 and E4) were stimulated with PMA or with PMA and fMLP, respectively, for 30 min in the presence of DHR, then immediately analyzed by flow cytometry (BD FACSCaliber). ROS generation is detected by oxidation of DHR into a fluorescent product and quantified as fold change (top right of each panel) in mean fluorescence intensity of DHR fluorescence (dark grey) compared with the unstimulated cells (light grey). Results shown are representative of two independent experiments.

For a quantitative assessment of the restoration of ROS activity, monocytes and macrophages were assayed using a DHR assay. This fluorescence-based assay relies on ROS converting the nonfluorescent DHR reagent into fluorescent rhodamine123b, providing a more quantitative measure of ROS production. As with the NBT assay, CGD1 and CGD2 cells showed little to no ROS production, whereas WT cells showed a dramatic shift in fluorescence upon stimulation (Fig. 4). The corrected single-cell clones, CGD2.GC16A.C4 and CGD2.GC16A.E4, phenocopy the WT cells both as monocytes and macrophages, and, as expected, the mixed population CGD2.GC16A has intermediate ROS levels. The lack of definition between positive and negative cells in CGD2.GC16A cells results from the very low levels of ROS activity in CGD2 cells (seen in both the NBT and DHR assays) and possible transfer of the ROS hydrogen peroxide from gene-edited cells to parental cells; addition of catalase to breakdown hydrogen peroxide minimizes, but does not completely remove, this effect [27].

**Figure 5:**
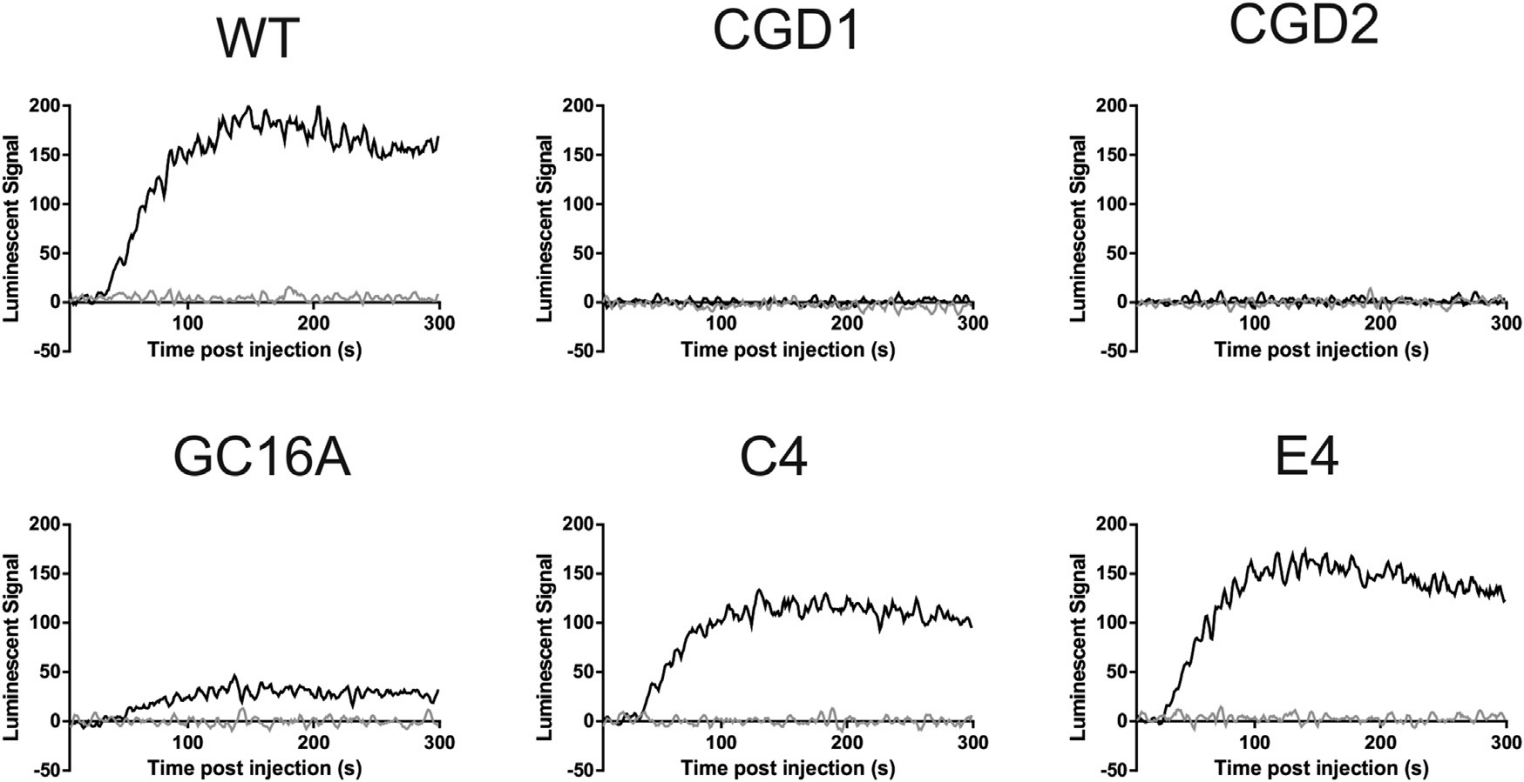
The kinetics of ROS production recapitulates WT cells after gene correction of *CYBB* in the CGD2 cell line. Monocytes from WT NHDF1 iPS cells (WT), a *P47^Phox^* mutant iPS cell line (CGD1), a *CYBB* mutant iPS cell line (CGD2), the mixed pool CGD2.GC16A of gene-edited iPS cells (GC16A), and the two gene-edited single-cell clones from CGD2.GC16A (C4 and E4) were exposed to luminol reagent in the presence (black line) or absence (grey line) of PMA stimulation, and individual wells were monitored for light released at 1 sec intervals for 300 sec in triplicate using a PHERAstar FS (BMG Labtech). The mean of three wells were normalized to the average of the 5 sec before luminol addition and are plotted with smoothing for clarity (average of four neighboring data points). Results shown are representative of three independent experiments.

Finally, to provide kinetic information about ROS generation, a real-time luminol assay was performed on the monocytes. Upon oxidation of the luminol reagent by ROS, light is released, which can be measured within a minute after addition of the cell stimulant PMA. As with the previous results, CGD1 and CGD2 showed no production of ROS upon stimulation, whereas clonal cells CGD2.GC16A.C4 and CGD2.GC16A.E4 showed similar quantities and kinetic properties to WT cells (max: WT = 169.5, C4 = 112.4, E4 = 147.4; slope: WT = 13.7, C4 = 12.96, E4 = 11.85; and time to reach 50% total signal: WT = 62.7 sec, C4 = 59.6 sec, E4 = 53.6 sec; sigmoidal curve fit) after the addition of PMA, and the mixed population CGD2.GC16A had lower levels but similar kinetics (max = 29.6, slope = 13.3, time to reach 50% total signal = 67.6 sec; Fig. 5).

### Lack of ROS in CGD2 cells is due to exon skipping, resulting in the absence of a functional protein

**Figure 6:**
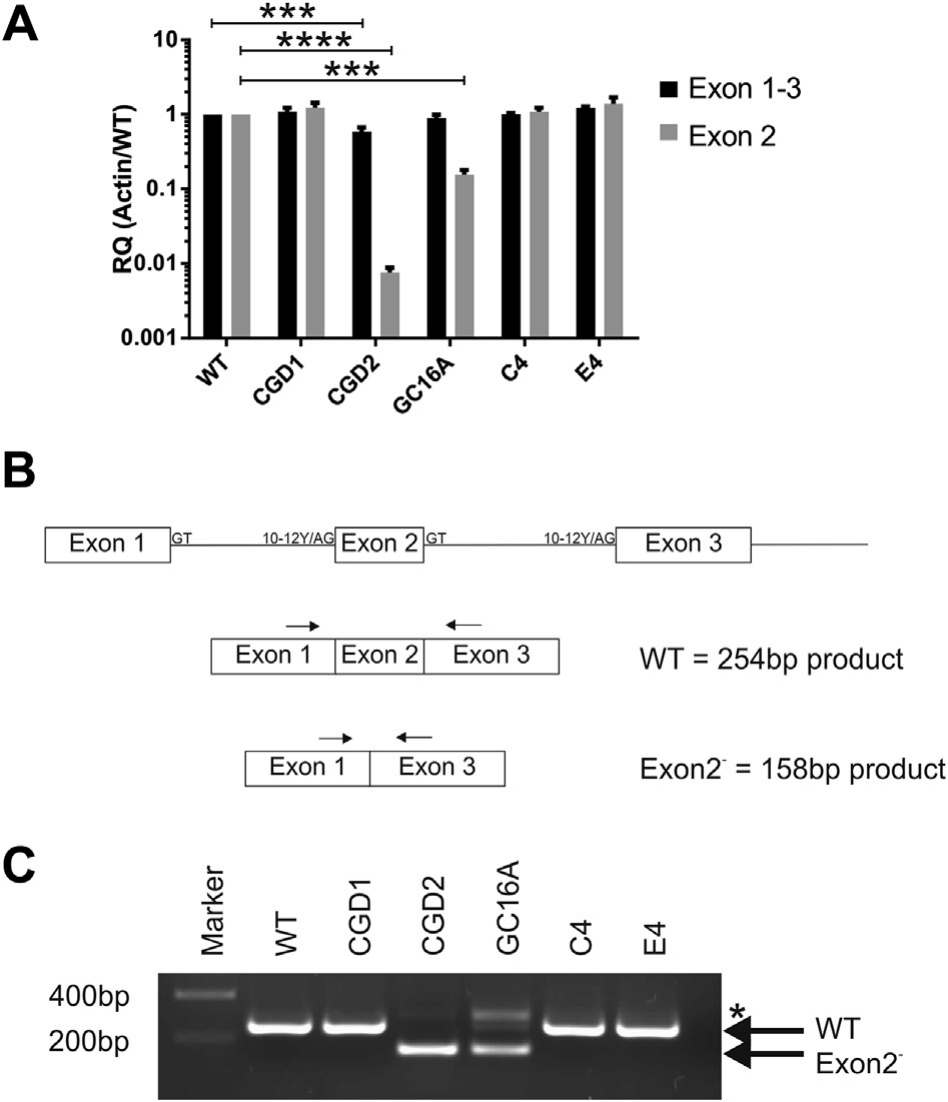
A single point mutation in the 3′ splice site of *CYBB* results in exon skipping. (**A**) RNA levels of *CYBB* from monocytes from WT NHDF1 iPS cells (WT), a *P47^Phox^* mutant iPS cell line (CGD1), a *CYBB* mutant iPS cell line (CGD2), the mixed pool CGD2.GC16A of gene-edited iPS cells (GC16A), and the two gene-edited single-cell clones from CGD2.GC16A (C4 and E4) were measured by qRT-PCR with two primer sets: one pair in which the primers bind the spliced junctions of exon 1-2 and exon 2-3 (Exon 2) and one splicing independent pair that binds exon 1 and exon 3 (Exon 1-3). Results are calculated relative to β-actin internal control primer pair, normalized to the WT cell line, and represent the average ± SEM of three independent cell harvests. Statistical significance was calculated by two-way analysis of variance with Sidak's multiple comparison test. ***p < 0.001; ****p < 0.0001. (**B**) Schematic representation of the *CYBB* mRNA pre- and postsplicing events producing either the correct splicing pattern (WT) or potential exon-2-skipped variant (Exon2^-^); the sizes of amplicon expected with primers binding exon 1 and exon 3 are shown. (**C**) qRT-PCR products using primers in exon 1 and exon 3 on cDNA from monocytes were separated on an agarose gel. Major bands corresponding to correctly spliced (WT) and the exon-skipped variant (Exon2^-^) are marked with an arrow. Larger bands in CGD2 and CGD2.GC16A (marked with an asterisk) were PCR artefacts.

**Figure 7:**
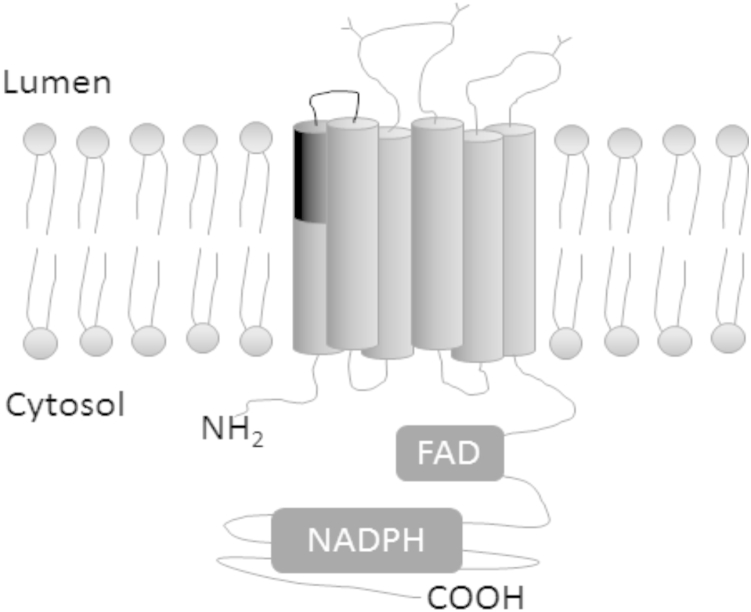
Location of exon 2 of *CYBB* when inserted into the membrane. Schematic representation of the CYBB protein within a membrane; the region encoded by exon 2 that would be lost due to exon skipping is highlighted in black.

Adapted from Marques et al. [49], based on Taylor et al. [50].

The cause of the ROS defect within CGD2 cells has not, to our knowledge, been previously investigated, so we took advantage of the isogenic properties of clones C4 and E4, which are genetically and phenotypically WT at the *CYBB* locus but within the genetic background of CGD2 cells, and we measured the levels of CYBB mRNA and protein in monocytes. Quantification of mRNA using primers located within exon 1 and exon 3 showed similar levels of CYBB mRNA across all cell lines, with CGD2 cells having a slight, yet significant, reduction (Fig. 6A). However, when using primers specific to the splice junctions of exons 1, 2, and 3, a more dramatic, hundredfold reduction in CYBB message in CGD2 cells was observed (Fig. 6A). These data indicate that, although CYBB mRNA is produced within CGD2 cells, it is incorrectly spliced. A splicing defect was confirmed by running the product of the exon 1 and exon 3 primers on a gel to observe the amplicon length compared with its predicted size (Fig. 6B). Correct splicing of the CYBB mRNA produces a product of 254 bp as observed (and confirmed by sequencing) in WT, CGD1, and the fully corrected CGD2.GC16A.C4 and CGD2.GC16A.E4 clones (Fig. 6C). In CGD2 cells, the WT product was not detected; instead, a smaller product of 158 bp was obtained, corresponding to an exon-2-skipped variant, the identity of which was confirmed by sequencing (Fig. 6C). The mixed population CGD2.GC16A showed both the exon-2-skipped variant band as well as a faint WT band from the corrected mRNA. An additional larger species, detected in CGD2, more pronounced in CGD2.GC16A, but absent in all other reactions, was found to be an artefact of the PCR reaction of a mixed population (in CGD2, 1% of the CYBB mRNA are correctly spliced according to the qRT-PCR results with splicing-specific primers in Fig. 6A). The CYBB protein is an integral membrane protein with six transmembrane domains, and the deletion of exon 2 results in the removal of half of the first transmembrane domain and the neighboring loop (Fig. 7), which is likely to lead to an incorrectly folded protein susceptible to endoplasmic reticulum (ER)-associated degradation or a nonfunctional, possibly topologically-altered protein.

## Discussion

Even in the era of potent antibiotics and fungicides, the diagnosis of CGD implies lifelong health complications and a reduced life expectancy [22]. In this article, we have demonstrated the ability of the CRISPR-Cas9 system to provide a footprintless strategy to correct a CGD-causing mutation at potentially therapeutically useful levels. This serves as a proof-of-principle in the development of genetically clean gene therapy approaches to cure this monogenic inherited disease.

We have shown here that the CRISPR-Cas9 system has the potential to provide highly efficient gene editing at the *CYBB* locus. This type of phenotypic correction is preferable to previous attempts to introduce the WT copy of the gene into iPS cells using lentiviral vectors [28] because it is completely clean; no residual foreign exogenous DNA remains to contaminate the genome and cause insertional mutagenesis, a hallmark problem with viral insertions. Moreover, the newly corrected gene is endogenously and, therefore, correctly controlled. This has been a major limitation to lentiviral introduction of exogenous transgenes, which either quickly become silenced or are not truly expressed in a cell-specific manner, despite numerous attempts to obtain specificity [29–31]. The limitation of nonendogenous control is also true of the recently published gene therapy strategies for CGD using zinc finger nucleases targeting the *AAVS1* safe harbor site for transgene insertion in iPS cells, in which WT copies of the mutant genes are constitutively expressed and, therefore, not myeloid-specific [28,32,33]. Targeting the causative mutation within the endogenous locus using scarless, or footprintless, gene editing is, therefore, the ideal gene therapy approach. Since high gene-targeting efficiencies are now possible with the help of site-specific nucleases, it just remains to transition this technology into clinically relevant cells for its potential to be fully realized. Although here we show correction of a single-point mutation that causes CGD, there are numerous mutations within the *CYBB* gene, as well as within the *CYBA*, *NCF1*, *NCF2* and *NCF4* genes, that are also responsible for CGD in different patients. Therefore, the strategy outlined here will require tailoring specific gRNA pairs and donor templates for each patient. It is worth noting that some genetic alterations within these genes may not lend themselves to CRISPR-Cas9 targeting (e.g., *LINE1*-retrotransposition) [34].

There are two methods that can be envisioned to exploit this technology therapeutically. The most tractable approach would be to convert the experimental protocol outlined in this article to hematopoietic stem cells (HSCs) isolated from bone marrow [35]. The technical limitations for such an approach would be the efficiency of gene editing in the HSCs and maintaining the bone-marrow-reconstituting potential of these fragile cells ex vivo throughout the transfection, expansion, and selection procedures. Alternatively, a technological advancement could allow for the creation of authentic bone-marrow-repopulating HSCs from iPS cells [36–38]. This would complete the circle of personalized medicine: patient-derived iPS cells for gene editing, expansion, and selection, differentiated into HSCs for reintroduction into the patient to repopulate the hematopoietic system with disease-free cells. Such a procedure, although theoretically possible, currently has multiple practical, safety, and ethical issues. Most importantly, the karyotypic stability of the cells would need to be very closely monitored. This becomes acutely obvious as more publications demonstrate the potential for stem cells in culture to accumulate mutations and karyotypic abnormalities over time [39–43]. Indeed, although the two single-cell clones isolated in this study were grossly karyotypically normal at the resolution of single nucleotide polymorphism (SNP) densities, this does not provide genome-wide sequencing levels of coverage [40,44], and a third clone from CGD2.GC16A that was karyotyped during the course of this study had an isochromosome 12p, a chromosome that has previously been seen to enhance the proliferation of stem cells in culture when duplicated [43]. Additionally, off-target effects of the CRISPR-Cas9 system are a potential issue, particularly if the cells are intended for clinical use. Although Cas9 uses complementarity between the gRNA and the target DNA to determine cleavage site selection, specificity is not absolute [45,46]. It was for this reason that we opted to use the nicking version of Cas9, which minimizes this issue [25,47]; however, clinical use may still require full genome sequencing to ensure the genetic integrity of the cells after gene correction.

Although neither of the potential clinical approaches outlined above is currently practicable, it is worth noting that site-specific nucleases have already been used ex vivo to modify T cells in clinical trials for human immunodeficiency virus (HIV), with no negative side effects [48]. Thus, further work is merited to transfer this technology into primary hematopoietic stem cells. Finally, a greater understanding of human hematopoiesis is necessary to generate repopulating HSCs ex vivo and thereby make personalized gene therapy a reality.
